# Implementation and preliminary clinical application of a robotic thoracic telesurgery program in a middle-income country: a structured pathway from simulation to clinical practice

**DOI:** 10.1007/s11701-026-03819-2

**Published:** 2026-08-31

**Authors:** Ricardo Mingarini Terra, Pedro Henrique Xavier Nabuco de Araujo, Marcos Naoyuki Samano, Fabio Eiti Nishibe Minamoto, Everson Luiz Almeida Artifon, Jose Pinhata Otoch, Paulo Manuel Pego-Fernandes

**Affiliations:** 1 Thoracic Surgery Division, Instituto do Coracao, Hospital das Clinicas HCFMUSP, Faculdade de Medicina, Universidade de Sao Paulo, Sao Paulo, Brazil; 2 Surgery Department, Faculdade de Medicina, Universidade de Sao Paulo, Sao Paulo, Brazil; 3 Thoracic Surgery Division, Instituto do Coracao, Hospital das Clinicas HCFMUSP, Faculdade de Medicina, Universidade de Sao Paulo, 2nd floor. Avenida Dr. Enéas de Carvalho Aguiar, 44, Sao Paulo, CEP 05403-000 SP Brazil

**Keywords:** Robotic surgery, Telesurgery, Thoracic surgery, Remote surgery, Health system implementation, Latin America

## Abstract

Telesurgery may help overcome geographic disparities in access to specialized surgical care. Although recent studies confirm the technical feasibility of robotic thoracic telesurgery, most reports originate from highly controlled settings, and practical implementation within public healthcare systems remains scarce. We describe the development and preliminary clinical application of a robotic thoracic telesurgery program in a middle-income country, reported as a proof-of-implementation case series rather than a statistically validated model. A robotic thoracic telesurgery program was developed connecting the University of São Paulo Medical School (FMUSP) and University Hospital (HU-USP), approximately 10 km apart, using the Toumai® platform over the institutional fiber-optic network. Development included infrastructure deployment, preclinical network validation (28 robotic surgeons), safety-protocol development, and team training, culminating in two remotely performed procedures under a pre-defined, manufacturer-limited case allocation. A robotic wedge resection for pulmonary metastasis and a robotic left upper trisegmentectomy with systematic lymphadenectomy were completed entirely under remote control, with the surgeon at FMUSP and the patient at HU-USP. Neither procedure had communication interruptions, packet loss, conversion events, or intraoperative complications; maximum latency did not exceed 12 milliseconds. Chest tubes were removed on postoperative day 1 in both patients, with discharge on day 1 and 2, respectively. This early experience supports the technical feasibility of introducing robotic thoracic telesurgery within a public healthcare system through a structured implementation pathway. Given the small sample, conclusions remain preliminary; the model’s value lies not in extending distance, but in outlining a reproducible process for expanding access to specialized surgical expertise.

## Introduction

Geographic disparities in access to specialized thoracic surgical care remain a major challenge worldwide. Although robotic thoracic surgery has expanded substantially over the last decade, expertise remains concentrated in a limited number of high-volume centers, and patients in geographically large countries frequently travel long distances to access complex thoracic surgical procedures.

Since the landmark Lindbergh operation in 2001, telesurgery has evolved from a proof-of-concept demonstration into a realistic strategy for delivering specialized surgical care across geographic barriers [1]. Advances in robotic platforms, fiber-optic communication, 5G connectivity, and network security have enabled the progressive transition from experimental studies to clinical application [2]. Recent reports have demonstrated the feasibility of remote robotic surgery in multiple specialties, including thoracic surgery, with procedures performed over distances ranging from a few kilometers to more than 8,000 km [2–4].

Thoracic surgery represents a particularly attractive field for telesurgical development. Experiences from China have demonstrated the feasibility of remote robotic lobectomy using dedicated 5G communication networks [3], while transcontinental robotic thoracic surgery has further expanded the boundaries of remote surgical care [4]. Nevertheless, most clinical experiences to date have originated from highly controlled environments, dedicated communication infrastructures, and specialized research programs, and the practical challenges of implementing telesurgery within real-world, resource-constrained healthcare systems have received comparatively little attention.

Brazil illustrates this challenge well: it has continental dimensions and a universal public healthcare system serving more than 200 million inhabitants, and specialized thoracic surgical expertise remains concentrated in a limited number of academic centers despite increasing adoption of robotic surgery. This conceptual rationale motivates our interest in telesurgery as a strategy to expand access to advanced thoracic surgical care. It is important to clarify from the outset, however, that the present report does not test telesurgery across continental distances: the two procedures described here connected a single institutional pair approximately 10 km apart. The relevance of this experience lies instead in the structured process required to move from infrastructure deployment to clinical application within a public healthcare system — a process that, in principle, is a prerequisite for any future extension to longer distances or additional sites.

International organizations have recently proposed frameworks and guidelines to support the safe implementation of telesurgery, emphasizing infrastructure validation, cybersecurity, surgeon credentialing, structured safety protocols, and progressive clinical adoption [5, 6]. However, reports describing the practical, end-to-end implementation of telesurgery programs within public healthcare systems remain scarce.

The objective of this study was therefore not to demonstrate telesurgery over continental distances, but to describe the development, preclinical validation, and preliminary clinical application of a structured robotic thoracic telesurgery program within the Brazilian public healthcare system, reported as an early technical feasibility and implementation-science experience rather than a statistically validated clinical model.

## Materials and methods

### Study design and ethical approval

This study was approved by the institutional ethics committee (CAAE 94,555,325.3.0000.0076), conducted in accordance with the Declaration of Helsinki, and is reported following the PROCESS 2025 guideline for case series. Written informed consent was obtained from both patients for the telesurgical procedure and for publication of clinical data.

## Program development

**Fig. 1:**
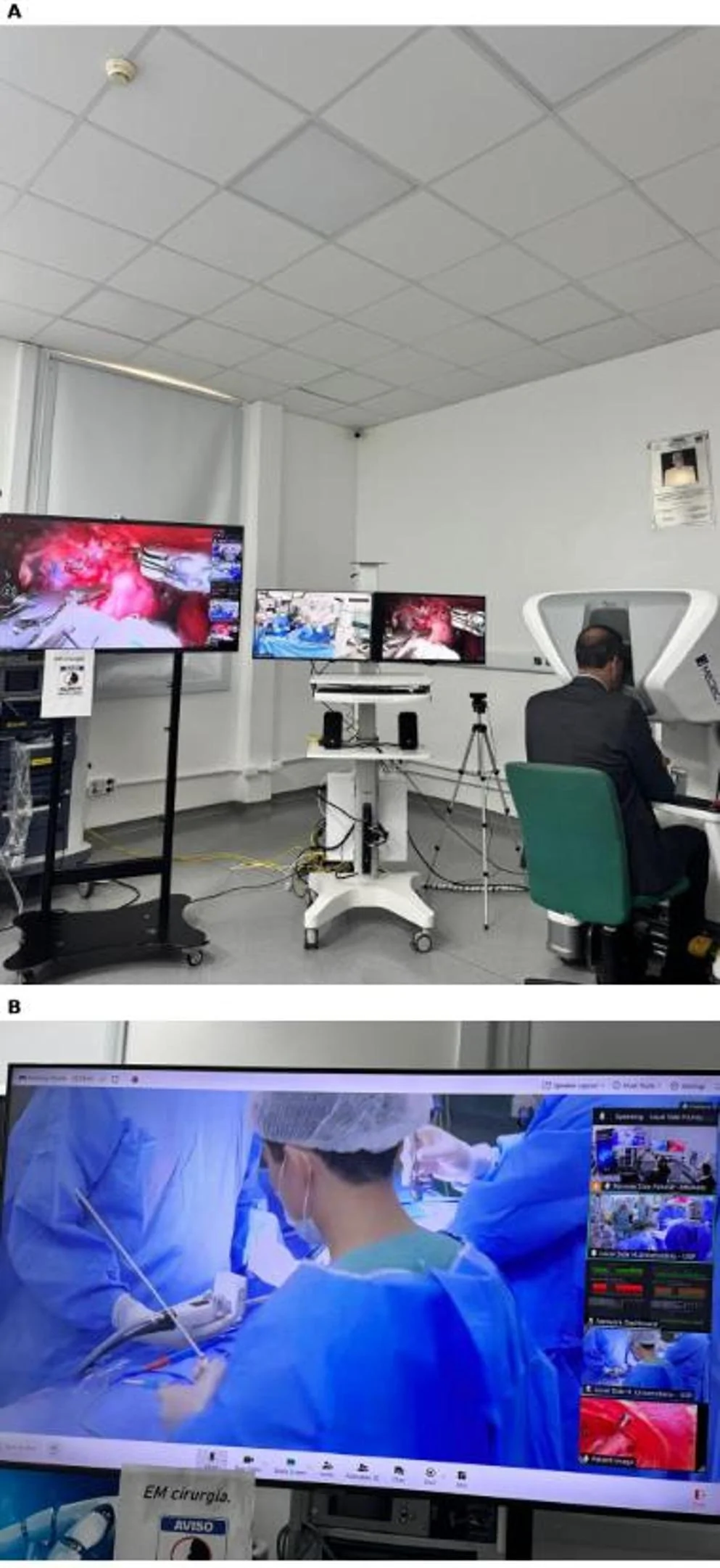
Remote surgeon console setup for robotic thoracic telesurgery. (**A**) Overview of the remote surgeon console suite at PROMIN-FMUSP, showing the operating surgeon at the Toumai^®^ console with dual auxiliary displays reproducing the endoscopic surgical field and the videoconference/network dashboard. (**B**) Real-time network operations dashboard during a telesurgery procedure, displaying the remote surgeon’s console feed (Remote Side, FMUSP-PROMIN), the local bedside surgical team (Local Side, Hospital Universitário-USP), live network performance monitoring, and the endoscopic patient image, viewed simultaneously by the local team

**Fig. 2:**
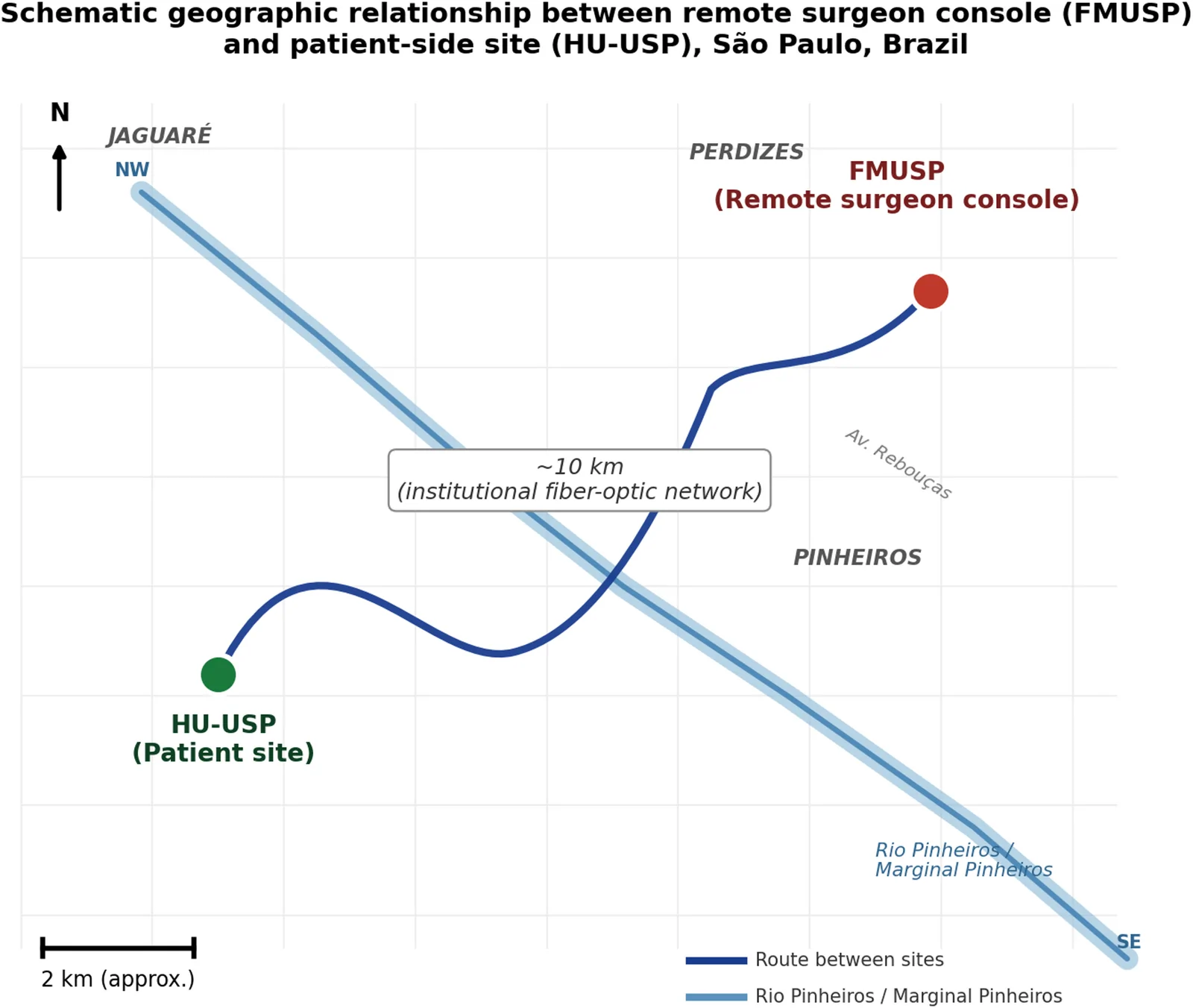
Schematic representation (not to exact geographic scale) of the physical separation between Faculdade de Medicina da Universidade de São Paulo (FMUSP), where the remote surgeon console was located, and the patient-side site (Hospital Universitário-USP), illustrating the approximate route distance (~ 10 km) connecting the two sites via the institutional fiber-optic network

The telesurgery program was developed through collaboration between the University of São Paulo Medical School (FMUSP) and the University Hospital of the University of São Paulo (HU-USP). We used the Toumai^®^ (MicroPort^®^, Medbot, Shanghai, China) robotic platform. The remote surgeon console was installed at the Center for Training in Minimally Invasive Procedures (PROMIN-FMUSP), while a second console and the robotic patient-side platform were located at HU-USP (Fig. 1). The two sites were connected through the institutional University of São Paulo fiber-optic network, separated by approximately 10 km (Fig. 2).

## Preclinical validation

Before clinical implementation, the platform underwent a structured validation process, reported in detail in a companion pilot study [7]. In brief, 28 robotic surgeons (23 tested under the institutional wired connection and 5 under a private 5G link, allocated according to scheduling availability) completed three standardized dry-lab tasks while latency, jitter, and packet loss were sampled approximately every 5 min from the robotic system’s built-in connectivity monitor. Wired sessions showed a median latency of 12.0 ms and median jitter of 6.8 ms, compared with 32.4 ms and 30.8 ms, respectively, under the private 5G configuration (both comparisons *p* < 0.001); median packet loss did not differ significantly between conditions (0.09% wired vs. 0.0% 5G, *p* = 0.096). Task success ranged from 82.1 to 100% across the three tasks, with all partial successes or failures occurring in wired sessions. Most participants rated the system as safe (78.6% assigned the maximum safety score) and suitable for clinical use (96.4%). These findings supported progression toward clinical application. Unlike the sampled monitoring used in this preclinical phase, the clinical procedures described below employed continuous, bidirectional monitoring of all network parameters (see Safety Framework). The two procedures described in the present study were performed using the institutional wired network connection; the private 5G network evaluated during the preclinical phase was not used in the clinical phase.

## Safety framework

A multidisciplinary safety protocol was developed before the first clinical procedure. Key components included:

- continuous, bidirectional monitoring of latency, jitter, and network stability, with dedicated information-technology teams stationed at both PROMIN-FMUSP and HU-USP, each managing connectivity troubleshooting for its respective site’s server;
- a maximum latency threshold of 200 milliseconds, consistent with the safety parameters recommended for robotic telesurgery in Brazil under resolution nº 2.311/2022 from the Federal Council of Medicine (CFM) [8];
- predefined conversion to local control in the event of network destabilization exceeding this latency threshold, any intraoperative technical or surgical event, or any problem identified at the local (HU-USP) console;
- experienced bedside thoracic surgeons capable of immediately assuming the procedure if necessary;
- uninterrupted communication between local and remote teams;
- institutional approval and dedicated informed consent.

This implementation strategy followed principles subsequently incorporated into recommendations from the Society of Robotic Surgery and the Japanese telesurgery guidelines [5, 6]. Because latency remained well below the 200 ms threshold throughout both procedures and no other trigger event occurred, the practical, real-time application of these predefined conversion criteria was not tested in this experience, which is discussed further as a limitation.

## Clinical application

The clinical phase of this program was conducted under a structured arrangement with the equipment manufacturer, which allocated a limited number of cases to each participating surgical specialty during the platform’s introduction; thoracic surgery was allocated two cases, which defined the scope of this report a priori rather than representing an open-ended program that was subsequently discontinued. Candidates were surgical patients with an indication for pulmonary resection amenable to a minimally invasive approach, who were selected as the first two eligible, consenting patients meeting this criterion following successful preclinical validation. Both procedures were performed entirely under remote robotic control from FMUSP with the patient at HU-USP.

## Results

### Network performance

Both procedures were completed entirely under remote control without communication interruptions, packet loss events, conversion to local control, or robotic-system incidents. Network parameters were monitored continuously and bidirectionally throughout both operations. Maximum latency did not exceed 12 milliseconds throughout both operations (Table 1).

**Table 1:** Evolution of clinical thoracic telesurgery compared with the first reported remote telesurgery experience

Study	Year	Country/Region	Procedure	Distance	Network Infrastructure	Reported Latency	Outcome
Marescaux et al.	2001	France–USA	Cholecystectomy	6,230 km	Dedicated fiber-optic network	Not reported	Successful
Tian et al.	2024	China	Right upper lobectomy	~ 5,000 km	Dedicated 5G network	~ 100 ms (average delay)	Successful
Gonzalez-Rivas et al.	2026	China–Romania	Single-port lobectomy	> 8,000 km	Dedicated 5G network	Not reported	Successful
Present study	2026	Brazil	Wedge resection and left upper trisegmentectomy	10 km	Institutional fiber-optic network	≤ 12 ms (maximum, bidirectional)	Successful

Unlike previous reports primarily focused on long-distance procedures, the present study emphasizes structured implementation within a public healthcare system. Latency values across studies were obtained under different network architectures and measurement methodologies and are presented for descriptive context rather than as a formal statistical comparison

## Case 1

A 21-year-old male with a history of high-grade malignant peripheral nerve sheath tumor developed a solitary 4-cm pulmonary metastasis in the left upper lobe following multimodal treatment. The patient underwent remote robotic-assisted wedge resection of the left upper lobe combined with en-bloc pleural resection and hilar lymphadenectomy. Total operative time was 106 min, with negligible blood loss. Network performance remained stable throughout the operation, with no packet loss, median latency of 11 ms, mean jitter of 10 ms, and mean packet loss of 0.1%. No communication interruptions, robotic-system incidents, or conversion events occurred. The postoperative course was uneventful; the chest tube was removed on postoperative day 1, and the patient was discharged home the same day. Final pathology confirmed metastatic malignant peripheral nerve sheath tumor.

### Case 2

A 47-year-old female presented with a slowly enlarging mixed ground-glass opacity highly suspicious for early-stage lung adenocarcinoma. The patient underwent remote robotic-assisted left upper trisegmentectomy with systematic mediastinal and hilar lymphadenectomy. Operative time was 140 min, with negligible blood loss. Network performance remained stable throughout the procedure, with no packet loss, maximum latency of 12 ms, and jitter ranging from 2 to 8 ms. No communication interruptions, robotic-system incidents, or conversion events occurred. The chest tube was removed on postoperative day 1, and the patient was discharged on postoperative day 2. Final pathology revealed lepidic adenocarcinoma staged as pT1aN0.

## Discussion

This report describes the implementation of a structured robotic thoracic telesurgery program within a Latin American public healthcare system, combining a preclinical network-validation phase involving 28 robotic surgeons with preliminary clinical application in two patients. Importantly, and consistent with the conceptual distinction drawn in the Introduction, the contribution of this experience does not lie in establishing a new distance record, nor in statistically validating clinical safety from two cases. Previous reports have already demonstrated thoracic telesurgery over distances exceeding several thousand kilometers: Tian and colleagues reported a successful ultra-remote robotic lobectomy performed over approximately 5,000 km using a dedicated 5G network, with an average delay of 100 ms [3], and Gonzalez-Rivas and colleagues described transcontinental single-port robotic thoracic surgery performed between Europe and China, although corresponding network latency data were not reported in the published abstract [4] (Table 1). Instead, our experience addresses a different, and complementary, challenge: the end-to-end process of introducing telesurgery into a real-world public healthcare environment, reported here as an early technical feasibility and implementation-science experience.

### Implementation science perspective

One of the most important findings of the present study is that successful telesurgery requires substantially more than a stable communication network. Program development involved sequential phases of infrastructure deployment, preclinical network validation, safety protocol development, multidisciplinary team training, and a pre-defined, manufacturer-limited clinical application. This structured pathway may be more relevant to future dissemination than the technical performance of the two individual procedures themselves, and may inform the design of similar programs at other institutions, although its reproducibility beyond this specific pair of sites has not yet been tested.

The staged implementation strategy adopted in the present study aligns with recommendations proposed by the Society of Robotic Surgery framework and the recently published Japanese telesurgery guidelines, both of which emphasize validation, credentialing, contingency planning, and progressive clinical adoption as fundamental requirements for safe implementation [5, 6]. A distinguishing aspect of our program was the use of objective preclinical validation before clinical application: in the companion pilot study, 28 robotic surgeons completed standardized dry-lab tasks under wired and private 5G conditions, with task success ranging from 82.1 to 100%, latency of 12.0 ms (wired) vs. 32.4 ms (5G), and predominantly favorable safety perception (78.6% maximum safety score) [7]. These preclinical findings, summarized in the Methods, provided objective evidence supporting progression to clinical application, though they were obtained in a simulated dry-lab setting and cannot substitute for clinical validation of the procedures themselves.

### Network performance and clinical feasibility

Both procedures were completed without packet loss, communication interruptions, conversion events, or robotic-system incidents. Network performance was continuously monitored in both directions throughout both operations via a dedicated dashboard (Fig. 1), with information-technology support stationed at both sites. Maximum latency did not exceed 12 milliseconds throughout all phases of both operations, well within the 200 ms threshold defined in our safety protocol [8]. This value is lower than the average delay of 100 ms reported by Tian and colleagues over a much longer distance using a dedicated 5G network [3], consistent with the shorter, wired connection used in our setting; comparable figures were not available for the Gonzalez-Rivas transcontinental report [4], and broader reviews of 5G telesurgery describe a range of latency profiles across different platforms and distances without a single directly comparable benchmark [2]. In addition to a pulmonary wedge resection, the platform successfully supported an anatomical left upper trisegmentectomy with systematic lymphadenectomy, demonstrating applicability to technically demanding thoracic procedures.

### Implications for Latin America

The implications of this model may be particularly relevant for Latin America, where robotic surgery continues to expand but high-volume thoracic surgical expertise remains concentrated in relatively few referral centers. Structured telesurgical networks may represent an opportunity to decentralize this expertise while preserving quality, safety, and educational standards, although realizing this potential will require moving well beyond the single short-distance institutional pair described here.

### Scalability considerations for middle-income public systems

Extending this pilot experience into a sustained clinical program would require addressing several practical challenges beyond network performance. These include the capital and maintenance costs of robotic platforms and dedicated connectivity within budget-constrained public systems; the absence of a specific regulatory pathway for telesurgery in Brazil, including questions of cross-institutional liability and licensing; cybersecurity requirements for protecting real-time surgical data transmitted between institutions; and the need for a structured training and credentialing pipeline for surgeons and bedside teams, distinct from conventional robotic surgery training. Several of these considerations echo those raised in the companion pilot study [7] and in recent international frameworks [5, 6], and are, in our view, at least as important to future dissemination as further technical validation of network performance.

### Future directions

The future impact of telesurgery may extend beyond direct patient care. The same infrastructure required for remote operations can support teleproctoring, telementoring, remote credentialing, and dissemination of advanced robotic thoracic procedures, as highlighted by recent telesurgery frameworks and reviews [2, 5]. Complex techniques such as anatomical segmentectomy, sleeve resection, advanced mediastinal surgery, and minimally invasive airway reconstruction could potentially be taught and supervised remotely, facilitating more equitable access to specialized surgical education. Regional telesurgical networks connecting academic centers and underserved hospitals may ultimately become one of the most important applications of this technology, though this remains a future direction rather than a demonstrated outcome of the present work.

### Limitations

This study has several limitations. The clinical experience is limited to two index procedures at a single institutional pair — a sample size that precludes any conclusion about generalizability, learning-curve effects, or rare adverse events, and that should be understood as a technical feasibility and implementation-science report rather than a validated clinical model. This sample size was not an interim or discontinued endpoint but reflected a pre-defined allocation of two cases to thoracic surgery under a structured, manufacturer-limited program; whether the program will be extended beyond this pilot phase has not yet been determined. The two sites were connected by an institutional fiber-optic network separated by a comparatively short distance (10 km) (Fig. 2), and network performance under this configuration may not directly extrapolate to longer-distance or public-internet-based deployments. The safety framework defined explicit conversion criteria (a 200 ms maximum latency threshold under Resolução CFM nº 2.311/2022 [8], intraoperative technical or surgical events, or problems identified at the local console); because no conversion event occurred in either case, the real-world responsiveness and timeliness of this protocol under actual degraded network conditions remains untested. Long-term oncologic and functional outcomes were not assessed. Finally, as with any early-phase implementation report, selection of index cases inherently favored technically straightforward candidates meeting minimally invasive resection criteria, which should be considered when interpreting the safety profile described here.

## Conclusion

This report describes a structured pathway for introducing robotic thoracic telesurgery within a Latin American public healthcare system, from infrastructure deployment and preclinical network validation to a pre-defined, manufacturer-limited clinical application in two patients. Given the small sample, this experience is best understood as an early technical feasibility and implementation-science report rather than a validated clinical model, and its conclusions should be interpreted accordingly. The value of telesurgery should not be measured by the distance between surgeon and patient, but by its ability to overcome disparities in access to specialized surgical expertise. As robotic surgery becomes increasingly available worldwide, telesurgery may evolve from a technological achievement into a healthcare-delivery strategy capable of reducing geographic disparities and facilitating access to advanced thoracic surgical care, provided the technical, regulatory, and training challenges outlined above are addressed in future, larger studies.

## Data Availability

No datasets were generated or analysed during the current study.
